# How Social Responses to Child Sexual Abuse and Intimate Partner Violence Affect Homelessness Among Women in Two Rural Regions With Resource-Based Economies in Eastern Quebec

**DOI:** 10.1177/10778012221083329

**Published:** 2022-06-09

**Authors:** Catherine Flynn, Pénélope Couturier, Simon Turcotte, Kim Dubé, Christophe Levesque, Philippe-Benoit Côté, Simon Lapierre

**Affiliations:** 1 Department of Social Sciences and Humanities, 14661 University of Quebec at Chicoutimi, Quebec, Canada; 2 School of Social Work, 6363 University of Ottawa, Ontario, Canada; 3 Center Urbanization, Culture and Society, 14851 Institut National de la Recherche Scientifique, Montreal, Quebec, Canada; 4 Department of Sexology, University of Quebec at Montreal, Quebec, Canada

**Keywords:** violence against women, childhood sexual abuse, intimate partner violence, homelessness, rural areas, feminist studies

## Abstract

This study presents findings from a qualitative study conducted in two relatively remote, primarily rural regions of the Canadian province of Quebec whose resource-based economic structures exacerbate inequalities between men and women. The purpose of this study was to understand how violence and homelessness intertwine in women's life courses in such regions. On the basis of past research showing that gender socialization around traditional roles and conservative values is particularly tenacious in non-urban areas, we conducted life-course interviews with 22 women in 13 different towns and villages of these two regions. Our content analysis of these interviews showed that specific social responses have forced women to maintain relationships with their aggressors or with people who have protected them, thus relegating these women's lives to the private sphere while reducing their opportunities for social participation in the public sphere. These social responses, together with women's economic and social disadvantages in these regions, were also the main factors that explain homelessness experienced by the participants in this study. Our analysis of these responses illustrates the patriarchal social structure of power in these regions, which is perpetuated in the interpersonal, institutional, and representational dimensions and keeps women in precarious, subordinate social positions, while ostracizing or punishing women who try to resist.

More and more studies have shown that in non-urban areas, gender socialization centered on traditional roles and conservative values is especially persistent, complicating the life courses of women who experience intimate partner violence (DeKeseredy et al., 2016; Farhall et al., 2020; Savard & Marchand, 2016). In Canada, few studies have been done on violence against women in non-urban areas, but the information that has been gathered on this subject is alarming. Compared with Canadian women in urban areas, Canadian women in rural areas, small towns, and villages are more likely to experience family violence (Sinha, 2013) and are overrepresented in recent statistics on femicide: Although only 16% of the Canadian population lives in rural, remote, or northern areas, 38% of women and girls killed in Canada in 2019 had been living in non-urban areas (Canadian Femicide Observatory for Justice and Accountability [CFOJA], 2020). It has been well established that many women in urban settings experience homelessness, especially when they try to escape violent situations (Broll & Huey, 2020; Kahan et al., 2020; Meyer, 2016; Milaney et al., 2020; Reid et al., 2021; Schwan et al., 2020; Sullivan et al., 2019; Tutty et al., 2013). But much remains to be learned about how the problems of violence against women and homelessness among women interact in non-urban settings.

This article presents findings from a qualitative study (Flynn et al., CRSH 2017–2019) conducted in two non-urban regions in the eastern part of the Canadian province of Quebec. These regions are classified by the Government of Quebec (2018) as “resource regions”: areas of low population density where various government policies promote the exploitation and export of natural resources as a means of economic development. In such regions, inequalities between men and women are exacerbated, but little research has been done on violence against women or homelessness among women. In this study, we sought a better understanding of how violence and homelessness become intertwined in women's life courses in such regions. The intersectional feminist analysis applied in this article shows how conservative values had structured social responses to the interview participants’ disclosures that they had been victims of violence, eroding their support networks and making their living conditions more precarious. Most importantly, this analysis reveals that the social responses that these women received from their relatives and from certain institutions had relegated these women to the private sphere, forcing them to maintain their relationships with their aggressors or with people who had shielded them, while reducing these women's own opportunities to participate socially in the public sphere.

This article begins with a review of the literature on violence against women and homelessness among women in non-urban areas. It then describes the main theoretical and methodological frameworks applied in this study. Next, this article presents the main findings from this study and brings them into dialogue with existing knowledge on these topics. Lastly, this article discusses the social power relationships that cut across the participants’ experiences and the various vectors by which these relationships are perpetuated.

## Distinctive Aspects of Violence Against Women in Non-Urban Areas

Feminist studies have theorized male violence as a strategy for disciplining women and keeping them in subordinate roles (Romito, 2008). DeKeseredy et al. (2016) found that in rural communities, the high prevalence of male violence against women is associated with the reproduction of male socialization centered on domination and traditional gender roles, which are reinforced by male peer support. At the same time, in such communities, the focus on family values and on women's role in maintaining family relationships places pressure on women to remain in the family setting even when they are experiencing violence from their intimate partners or other family members (Farhall et al., 2020; Savard & Marchand, 2016) While thus receiving scant support from their families, women living in sparsely populated areas cannot rely on neighbors to detect the violence that they are suffering and take steps to ensure their safety. In contrast with urban settings, geographic isolation reduces the chances that episodes of violence against women will be seen and heard and that women will have quick access to a resource or a safe place where they can seek help (Bourassa & Savoie, 2005; Dekeseredy, 2019; Savard & Marchand, 2016).

In small communities where many people have family, friendship and professional ties, anonymity and confidentiality are hard to come by. Hence, women who are victims of intimate partner violence may let feelings of shame, fear of being seen as “failures,” or fear of what other people will say keep them from reaching out to others for help and support (Farhall et al., 2020, p. 191). In such communities, fear of reprisals from abusers or their friends and family is also especially prevalent (Bourassa & Savoie, 2005; Farhall et al., 2020; Savard & Marchand, 2016). Moreover, representatives of the justice system, such as police officers, lawyers, and judges, often protect perpetrators with whom they have social ties, such as sports teammates and hunting and fishing buddies (Dekeseredy, 2019; Farhall et al., 2020).

Besides these issues of values and the way that community life is organized, rural areas have fewer public and community services than urban areas, and some resources are difficult to access or nonexistent (Dekeseredy, 2019; Farhall et al., 2020; Zufferey & Parkes, 2019). For example, compared with urban centers, rural areas have significantly fewer resources such as emergency shelters to support women and children who are victims of violence (Dekeseredy, 2019; Farhall et al., 2020; Savard & Marchand, 2016). Having to travel a long distance to seek a professional service or to obtain immediate help prevents many women from getting the support that they need (Bourassa & Savoie, 2005). Moreover, in the vast majority of rural areas, lack of public transportation poses a significant obstacle for women trying to escape from violent situations (Clennett-Sirois, 2015; Dekeseredy, 2019; Farhall et al., 2020; Savard & Marchand, 2016). In theory, modern information and communication technology might help improve access to services for women victims of intimate partner violence, but in many rural communities, Internet access and cell phone connectivity are limited (Farhall et al., 2020).

### Connection Between Violence and Homelessness Among Women and Girls

Violence is now well known to be the main factor explaining shifts into homelessness among women and girls (Gaetz et al., 2013). Significant risk factors for housing instability among women include neglect and sexual and physical abuse experienced in childhood (Broll & Huey, 2020; Jasinski, 2010; Laberge et al., 2000; Lewinson, Thomas & White, 2014), intimate partner violence (Murray, 2011; Spinney, 2012; Tutty et al., 2013), and sexual violence (Tyler et al., 2001). Broll and Huey (2020) found that adult victims of physical assault and those who have experienced “multiple forms of violence in childhood, adulthood, and across the life course are more likely to be homeless multiple times” (p. 3381), and that “for each additional form of victimization experienced [intimate partner violence, physical assault, sexual assault and gang-related violence], women's likelihood of experiencing multiple episodes of homelessness increases by about 36%” (p. 3393). Other authors identify the moment of escaping from a violent family situation or leaving an abusive partner as a critical one for women, where the risk of experiencing precariousness and housing instability is very high (Hamby, 2014; Jasinski, 2010; Meyer, 2016; Sullivan, Bomsta & Hacskaylo, 2019; Tutty et al., 2013). For some women, being homeless after fleeing untenable situations means that they have successfully applied a survival strategy and protected themselves from violence (Gélineau, Seck & Brisseau, 2008; Tutty et al., 2013).

In a study by Employment and Social Development Canada (2017), 25% of women cited intimate partner violence as the main reason for their most recent episode of homelessness. Meyer (2016) even suggests that most women who separate from abusive partners move into a homeless situation by definition: Most often, they leave their homes for unstable alternative accommodations with friends, family, or strangers or in emergency shelters (Meyer, 2016; Tutty et al., 2013). In Tutty et al. (2013), some victims of intimate partner violence reported having experienced homelessness while still living with their abusive partners; living in constant fear of being kicked out by their partners and having nowhere to go placed them in a state of housing precariousness. Some authors even argue that simply living in a violent, abusive situation constitutes housing instability by definition (Gregory, 2001; Robyn, 2001). The term “homeless with a shelter” refers to the experience of women who have a place to live, but whose living conditions threaten their safety (Robyn, 2001).

Violence creates various barriers for women trying to find stable housing when leaving abusive situations (Meyer, 2016; Tutty et al., 2013). Although violence affects women and families of all economic backgrounds, victims of violence generally experience financial insecurity, often following years of economic violence when their finances were monitored, controlled, or restricted (Hamby, 2014; Meyer, 2016). Many women find themselves at a disadvantage in their efforts to secure housing, and after staying in emergency shelters or with relatives, realize that they do not have the financial means to live alone. A definitive break with domestic violence is often preceded by multiple trial separations and attempts to seek help and resulting alternation between relatively stable housing and housing instability (Meyer, 2016; Tutty et al., 2013). According to Meyer (2016), a woman's choice to stay with, leave, or return to a partner represents the outcome of a careful decision-making process in which she weighs the availability of safe, sustainable housing solutions.

Few studies have been done on poverty and homelessness in rural or low-density areas in Canada (examples include MacDonald & Gaulin, 2020; Zufferey & Parkes, 2019), and much remains to be learned about this subject. Because of the many ways that women tend to hide their homelessness, they are still underrepresented in studies on this subject (Milaney et al., 2020; Speedlin et al., 2020), and as yet there is only limited feminist research documenting women's experiences of homelessness and problematizing this reality as the product of interpersonal and structural violence perpetrated against them (Milaney et al., 2020; Speedlin et al., 2020).

The main goals of the present study were to determine how violence and homelessness overlap in the life courses of women from two regions of the Canadian province of Quebec that are largely rural and have largely resource-based economies and to understand the structural processes involved when women in such non-urban areas experience situations of homelessness.

## Theoretical Framework—Intersectionality

This study was grounded in the intersectional feminist paradigm (Collins, 2000; Collins & Bilge, 2017; Crenshaw, 1990; Hancock, 2007), which takes an integrative perspective on domination, postulating that women such as those in this study face numerous forms of oppression that cannot be considered only separately or additively (Bilge, 2009, 2010; Collins & Bilge, 2017). Our premise was that by interviewing women who had experienced both violence and homelessness, we could document how these two social issues can occur at the intersection of various social power relationships linked to gender, socioeconomic status, physical and mental health, and age.

The intersectional paradigm originated in the critical anti-racist perspectives of Black feminism (Collins, 2000; Collins & Bilge, 2017; Crenshaw, 1990), and in the production of knowledge about violence against women and homelessness, it is important to avoid discounting BIPOC women's voices and whitewashing these experiences. In considering the representation of immigrant and Indigenous women in some parts of the two regions included in this study, it is necessary to examine how colonialism and racism are likely to intersect with these women's experiences.

Although these kinds of power relationships are often the subject of theory and analysis in studies on intersectionality, the experiences of participants in such studies may also be influenced by power relationships related to the urban/rural dimension, about which less is known. Some studies in critical geography have addressed the hierarchical relationship between large urban centers and outlying regions within the capitalist system (Brenner & Schmid, 2015; Merrifield, 2013) and shown how rural populations, and especially women and children in rural areas, are marginalized and constructed as altered groups (Little, 1999). It is therefore essential to examine how the positionality of these women in rural areas contributes to the construction of their experience. A sufficiently accurate, nuanced analysis of this kind should make it possible to grasp the internal diversity of this group and thus avoid reproducing stereotypical representations and patterns that homogenize the experiences of these women.

Among the various frameworks available for conducting multilevel analyses of interviews with marginalized women, the present research team chose one that the principal researcher has tested over time (Caron et Damant, 2015; Chbat et al., 2014; Flynn et al., 2014; Flynn et al, 2016). This analytical framework considers the reproduction of social relations of power in the following dimensions: representational (Yuval-Davis, 2016), institutional (Foucault, 1975), everyday-violence (Scheper-Hughes & Bourgois, 2004), and intersubjective (Yuval-Davis, 2016).

The representational dimension emerges in interview excerpts that portray the ideologies present in the dominant society and culture, and expressed by family members that placed added pressure on these women. This dimension is linked to the others, because it serves as the backdrop to the social relations of power reproduced in the other dimensions (Bourdieu, 1980).

The institutional dimension is illustrated in excerpts where participants discuss laws, policies, and practices that exclude or control them. It also incorporates their complicated interactions with health and social services institutions (Lagraula-Fabre, 2005) and the legal system (Campbell et al., 2009).

The everyday-violence dimension includes the manifestations of social power relations in day-to-day interpersonal relationships and interactions with intimate partners and other family members (Bilge, 2015; Collins, 2000; Scheper-Hughes & Bourgois, 2004). This dimension includes behaviors in which members of women's social or family circles seek to trivialize the violence that they are suffering or pressure them to suffer it in silence.

Lastly, this study also analyzed the intersubjective dimension, as reflected in excerpts in which the interview participants accept, reject, or negotiate specific social relationships, identifications, or roles imposed on them. Later in this article, we place our analysis of this dimension in dialogue with that of the representational dimension.

## Methodology

The content analyzed in this study came from 22 qualitative interviews, based on the life course approach (Cavalli, 2007), that were conducted with women from Bas-Saint-Laurent and Gaspésie-Îles-de-la-Madeleine, two regions of the Canadian province of Quebec that are largely rural and have largely resource-based economies. Reports by Quebec's Council on the Status of Women (CSF, 2015a, 2015b) show that gender inequalities persist in these two regions, but these inequalities are often overlooked in Quebec research on homelessness among women and violence against women in these regions. For example, in these regions women generally have higher educational levels than men but also have a higher unemployment rate and are more likely to have low incomes. In general, motherhood can produce disruptions in women's career paths, but in these regions women are especially underrepresented in the most vital sectors of the local economy. Furthermore, as the population of these two regions ages, the proportion of older individuals living alone is higher among women than among men. In these two regions, women are twice as likely as men to rent their homes (CSF, 2015a, 2015b). In response to this situation, in Fall 2016, an awareness campaign to fight violence against women renters in these regions was carried out by a Quebec housing-rights organization (the Front d’action populaire en réaménagement urbain [FRAPRU]) and a Quebec women's advocacy group (the Centre d’éducation et d’action des femmes de Montréal [CÉAF]). Recent data also show that in Bas-Saint-Laurent and Gaspésie-Îles-de-la-Madeleine, intimate partner violence accounts for half of all crimes reported to the police, and that 80% of the victims in these cases are women (CSF, 2015a, 2015b). The Gaspésie-Îles-de-la-Madeleine region ranks fourth out of 17 in Quebec for prevalence of intimate partner violence.

### Recruitment of Interviewees

To be included in this study, during the five preceding years, women had to have experienced violence at the hands of a family member, intimate partner or third party and at least one situation of homelessness (such as having stayed with family, having stayed one or more times at a women's or emergency shelter, having had difficulty in paying rent, or having been out on the street). Women were recruited from December 2017 to December 2018 from women's support and housing services specializing in homelessness, intimate partner violence, sexual assault, feminist intervention, and mental health in the regions of Bas-Saint-Laurent and Gaspésie-Îles-de-la-Madeleine, Quebec. These regions are quite large, and these services are scattered quite widely throughout them, so the recruitment effort covered an extensive geographic area. Participants were interviewed in 13 different small towns and villages in these two regions, in order to avoid collecting data solely from their urban centers.

### Data Collection and Analysis

The life-course interviews varied in length from 2 to 4 h, depending on the participants’ experiences and openness. Working with the interviewer (always a woman), each participant filled out a calendar of her life course, identifying key events related to violence and homelessness. The interviews covered various topics, such as the forms of abuse that the participants had suffered, the various ways that the people around them had reacted to it, the episodes of homelessness associated with these experiences, the strategies that these women had developed to survive and to protect themselves, the obstacles that they had encountered when applying these strategies, their approaches to requesting help, and other subjects. The interviews also captured other significant aspects of the participants’ life trajectories, including their physical and mental health, motherhood experiences, substance use, and paths to social and professional integration. These interviews were transcribed, anonymized and diagrammed using *frise chronologique*, a timeline-creation tool available at www.frisechronos.fr. They were then subjected to a mixed thematic content analysis (L'Écuyer, 2011) structured around the four dimensions described above (representational, institutional, everyday violence, and intersubjective), as well as through analytical categories that emerged from the participants’ stories. This analysis was performed using NVivo 12 software.

### Description of the Sample

At the time of the interviews, the 22 participants ranged in age from 23 to 61—a range that has proven effective for documenting age-related social power relations. Of the 22 women interviewed, 16 discussed their experience of motherhood, which will be the subject of a forthcoming master's thesis. Although physical health was not a priority topic in the current study, 10 of the participants described temporary or permanent disabilities that they had experienced: three since birth, two as the result of accidents, two as the result of violence, and three as the result of chronic diseases that they attributed either to their harsh living conditions or to earlier experiences of violence. As discussed in another article (Godin & Flynn, forthcoming), these 10 participants’ discontinuous trajectories in the labor market (all of them as domestic workers) illustrate how their precarious living conditions were created by the overlapping effects of sexism and ableism—the same forces that created dependency on intimate partners who used their privileged positions to dominate these women.

Nine of the interviewees also discussed their mental-health status. All nine had been diagnosed with depression, while two had also been diagnosed with bipolar disorder, one with post-traumatic stress disorder, and one with borderline personality disorder. Five of the interviewees had engaged in illicit activities (such as drug dealing), had been imprisoned, or both, and three more spoke about their experiences with prostitution or other aspects of the sex industry. The research team recognizes that it would have been desirable to include more women from various marginalized groups, but for reasons about which we hypothesize later in this article, almost all of the women recruited in the sample were White and cisgender. The only exceptions were one Indigenous (Métis) woman and one trans woman.

## Results

All 22 of the participants had made a transition to homelessness at some time in their lives: nine of them at the end of adolescence (often to escape a violent family situation, such as having been sexually, physically or psychologically abused by a family member or having witnessed domestic violence), and the 13 others at the end of a long, significant romantic relationship. One major finding from the interviewees’ life stories is that beyond the traumas and consequences of violence, their transition to homelessness was attributable mainly to lack of support and acknowledgement of their experiences from the people around them and from the various institutions and organizations from which they had sought help after fleeing violent situations. Several of the participants said that when they told members of their social networks about abuse that they experienced as adolescent girls or adult women, or reported it to the authorities, no one took any action to protect them. This lack of social and institutional recognition and support drove the participants into increasing economic insecurity and eventually episodes of homelessness.

In the first of the following three sections, we present the interpersonal dimension of the participants’ experiences: their descriptions of the reactions of family and friends whom they told about the abuse that they were suffering. In the second of these sections, we describe some of the institutional responses that tended to delegitimize these women's voices and keep them in situations of violence or homelessness. In the third section, because many of the participants’ narratives revealed values and discourses prevalent in their families and communities with regard to the roles of women and men, we analyze the representational dimension. And because some of the participants were highly critical of these values and discourses, we place this representational dimension in dialogue with the intersubjective dimension. Some of the participants rejected the roles assigned to them, while others resigned themselves to conforming to them, especially in situations where they faced significant economic and social constraints.

### The Interpersonal Dimension: Friends and Family Who Deny and Trivialize Violence and Make it Invisible, Victims Who Persevere and Resist

Our analysis of the participants’ life-course interviews showed that disclosing lived experiences of violence to their loved ones had proven incredibly painful, especially when the perpetrators (almost all of them men) were family members. This is a troubling finding, because family and friends are the primary sources of support for women who experience abuse (Trotter & Allen, 2009). Eight of the 22 women in our sample said that family members and friends whom they told about experiences of violence believed them but usually did not provide the necessary support to keep them safe. Further analysis of the interviews identified a total of 50 references showing that 16 of the 22 participants had at one time or another had to deal with relatives who had denied the alleged violence (six references), minimized or trivialized it (nine references), abetted or been manipulated by the abuser (seven references), supported him (14 references), refused to listen and thus took no responsibility to provide support (six references), or blamed the women themselves for the violence that they had suffered (eight references).

Among the 11 participants who said that they had been sexually abused in childhood (61 references), some, such as Jenny, said that when they reported their versions of the facts, they were met with doubt. For example, Jenny's father did not believe her when she recently confided in him about two sexual assaults committed by a member of her family when she was a child, an experience from which she still carries the trauma today.

A couple of months ago, when it all really hit me, it really came back to me. I told my parents (…) Dad didn’t believe me. I’m the one at fault because I’m talking about it after so long, I should just let it go (…) my grandpa tried to … he touched me … he tried to kiss me and he was touching me and was putting his hands in my pants. It's gross … Gross … And I told him “grandpa did this” and he didn’t believe me, he got angry at me! Ahhhh fucked up family … fucked up family …! (Jenny, Îles-de-la-Madeleine)

Other participants told how people around them who knew about their experiences of violence and abuse treated them as something private or to be kept secret. For example, many people in Marie's community knew about her father's controlling, violent behavior, but when she asked neighbors for help on one occasion when he became especially violent, here is how they reacted:

I’ll tell you, we lived in a small town, in (name of town). It's about 25 minutes from (name of city). Everybody knew each other, but no one spoke up, and it frustrated me! Everybody knew, for sure when you were violent. … They didn’t talk (there was just) one time when I went outside, I was looking around at people because the neighbors were outside and a lot of stuff was happening: “What are you doing?” I was yelling. I was saying, “Call the police, do something.” … “It's none of our business. We won’t get involved in this.” (Marie, Gaspésie)

Another participant, Carole, believed that her grandmother had tried unsuccessfully to protect her from being sexually abused by her grandfather when she was 4 years old, but that her mother blamed her for what had happened and warned her not to talk about it:

I didn’t talk about it at the time because it's true that I knew that if I talked … It wasn’t him, I was the guilty one. It was my fault if it happened. … But I couldn’t talk about it because it was the family secret and if I did there would be hell to pay. (Carole, Bas-Saint-Laurent)

In discussing the ways that victims of intimate partner violence are stigmatized, Overstreet and Quinn (2013) describe the concept of cultural stigma: societal attitudes and prejudices whereby victims are judged and blamed and their experiences are trivialized and delegitimized. Some of our participants’ comments about how their disclosures of intimate partner violence were received show the kind of reasoning by which cultural stigma operates. Johanne, for example, described how her siblings failed to understand the cycle of intimate partner violence and instead blamed her for returning to her abusive spouse so many times:

Even my brothers and even one of my sisters have said to me before: “You must like it! You keep going back.” (Johanne, Gaspésie)

In another example of cultural stigma, when Louise, who lived in a fishing community, tried to talk about the physical and psychological violence that she had suffered at the hands of her intimate partner, her family members tried to rationalize her experiences as a natural consequence of the stresses and strains of the fishing life.

People said that it was just a fishing fight. No, it wasn’t just a fishing fight. (Louise, Îles-de-la-Madeleine)

For some of our participants, trivialization of the violence that they had experienced was especially difficult when it came from their adult children. In Louise's case, the violent husband whom she had left was a prominent member of the local fishing community. Her son enjoyed the social and economic status that his stepfather's position gave him and so blamed his mother for having provoked his violence, as she describes here:

And he was saying: “Get back with your ex! Why did you leave him? It's you, you make lots and lots of trouble and” … I said: “Well he called me all kinds of names, he pushed me” (her son answered) “It doesn’t matter!” It was really hard. Really! (Louise, Îles-de-la-Madeleine)

Another interviewee, Gisèle, had been living with a disability since birth. Her husband had coercively controlled every aspect of her life for over 20 years, but she had stayed with him to keep their family intact, finally leaving him when their daughter grew up and went away to college. Gisèle did not want to talk much about her relationship with this daughter, who still does not forgive her for leaving the marriage. Regarding this daughter, Gisèle says:

She makes me pay dearly … (becomes emotional) … for ending the relationship with her dad. (…) She doesn’t want me in her life anymore. So I find that hard. (Gisèle, Gaspésie)

The preceding interview excerpts are just a few of the many instances in which participants described how they felt misunderstood, blamed, or punished for the violence that they had suffered. Their stories showed that delegitimization of victims’ experiences (Overstreet & Quinn, 2013) were not limited to cases of intimate partner violence; it also included cases of child maltreatment and childhood sexual abuse. The participants told how their relatives had failed to recognize what they were going through and had betrayed ignorance about the various forms of violence perpetrated against women and the consequences that it can have. Even when the victims’ relatives did listen to and acknowledge their stories of abuse, they then often trivialized them and treated them as personal matters that should be kept secret and private.

Together with the inadequate institutional responses described in the following section, these societal reactions to violence and assault undermine women's living conditions and lead them into homelessness. For example, our analysis of the participants’ life courses shows that a negative experience with disclosing childhood sexual abuse can mark the start of a trajectory that leads to homelessness. Our analysis also shows that the time when participants disclosed the violence that they had suffered was also the time that they were at greatest risk of a decline in their living conditions. Participants’ comments reported in the preceding pages also show how the voices of young women in particular were not valued and heard. On the contrary, they were perceived as subversive, because they revealed how prominent men in these communities with their resource-based economies misused their power over women. The participants’ stories show how attempts were made to invalidate the experiences reported by young women, in what can be seen as the intertwining impacts of sexism and ageism on their life courses.

### The Institutional Dimension: Institutions and Organizations Whose Responses Relegate Violence to the Private Sphere and Force Victims to Maintain Family Ties

Many of the abused and disadvantaged women interviewed in this study said that public institutions and community organizations had given them valuable support and helped them to cope when their family and friends did not. But many of these women also described numerous obstacles that had complicated or limited their access to such resources. In some cases, when participants went to such resources for help in coping with violence or stabilizing their living conditions, they instead received responses that reinforced the discourse of their friends and family—the idea that abuse was something to be kept personal or private, something for which these women themselves were somehow to blame. In other cases, the social and economic structure of their communities as a whole excluded women whom we interviewed from the main spheres of social participation, undermined their living conditions, or forced them to fall back on family members for support.

Research on violence against women in rural settings has shown that these women have limited access to various support resources (DeKeseredy, 2019; Farhall et al., 2020). Women in the two rural regions considered in this study are no exception: 13 of our participants explained in their interviews (24 references) that people who live in remote parts of these regions have to travel long distances to communities where various resources and services are available. As in many other parts of the province of Quebec, these community resources are overworked and underfunded and sometimes struggle to meet their users’ needs (Schwan et al., 2020). This problem has been aggravated by two major provincial-government initiatives to consolidate community health and social services, first in 2003 and then in 2014, which saw many local community health and social-service centers closed and most services centralized in urban areas (Bolduc, 2017). Other studies that we are currently conducting show that these restructurings of social services have forced many organizations to set priorities for who will receive assistance. Fifteen of the women in this study said that they had been refused services unfairly or received inappropriate referrals (98 references), which in some cases had forced them to remain in violent situations or bear the blame themselves for the abuse that they had suffered.

For example, Diana, who had experienced violence many times in the course of her life, was working with a community resource person whom she trusted very much. But her needs and requests became so overwhelming that this person ended up exaggerating her symptoms in order to get her a stay on a psychiatric ward.

I felt betrayed. Because it was going well! (…) They sent me to the hospital, to the psychiatric ward (sobbing), the psychiatric ward! I was locked up in a room for two days (sobbing) I think it was the worst thing in my life. (…) I tried to tell them that it didn’t make any sense. (…) I didn’t trust the system anymore. (Diana, Bas-Saint-Laurent)

Louise and her adult son lived together in low-rent housing. When he ransacked their home, she tried to get admitted to a shelter, but they turned her down and told her that she should be able to throw her son out and ensure her own safety in her own home.

I was refused. I called (name of the resource) and they told me no because I was at home. And my son needed to leave. I should put my foot down. […] It didn’t work … (Louise, Îles-de-la-Madeleine)

Louise also experienced another problem that has been well documented in smaller communities, especially in situations that require confidentiality: the people to whom victims of violence turn for help often have family, social, or professional ties with the other parties involved (Bourassa & Savoie, 2005; DeKeseredy, 2019; Farhall et al., 2020; Savard & Marchand, 2016). Thus, when Louise went looking for help, she ran into problems because some of the people she turned to had various ties with her son or her ex-partner and perceived her as an outsider, which she believes led them to question her word about the violence that she had suffered at their hands. Louise also said that members of a self-help group that she had joined refused to listen to her experience of intimate partner violence because her ex-partner was a well-known, well-respected man in her village.

Even when we talked, when we went around the table, I wanted to talk about it, but some of them said to me, “You know, we know him, we can’t talk about someone we know. (…) You don’t have any business telling us that. It doesn’t concern us. It's none of our business. And it's men.” So … I couldn’t talk about it. So I felt, I asked myself if (name of the group) was worth it. (Louise, Îles-de-la-Madeleine)

In theory, a community's police force is responsible for ensuring the safety of women and children who are victims of violence, but 27 references in our interviews show that some participants felt that the police had not protected them well enough:

When I separated, I used to go see the police often. And they would tell me, “Well we can’t do anything!” They gave me tips: change your phone, change your phone number, change this and that … (Nathalie, Bas-Saint-Laurent)

Some other important points regarding the police: one interview participant said that there was no police service in her village, while other participants who reported positive experiences with the police either had family contacts in the police department or had been abused by someone who had been convicted of abuse before. When one participant called the police in a situation where she feared for her life, she was deemed to have violated the terms of her parole, which required her to stay at home and refrain from disturbing the peace, and she was therefore sent back to prison. As she explained:

The situation degenerated. I called the police because he strangled me, and I thought I was going to die. It turned against me because it was a breach of parole. (…) I was under certain conditions at his place, so I had broken the peace. Everything turned against me that time. I was sent back to prison … (Joannie, Bas-Saint-Laurent)

Thus, in addition to being blamed and punished for the violence she had suffered, Joannie was actually sent back to prison and thus deprived of social participation and access to the public sphere.

The interviews showed that even when participants did not face incarceration as Joannie did, their social and economic integration was often compromised by the presence, trivialization, or tolerance of violence as well as by lack of support and limited job opportunities in geographic areas where economic activity is concentrated or the population is small. Suzie told how, before her husband was incarcerated, she used to fear running into him in public places:

You know, sometimes I went to the hospital with the little one, and I was scared to see him there. I was scared. When I went into stores, I was scared. (Suzie, Gaspésie)

Other participants saw their employment relationships weakened because of their abuser's presence in their workplace. Hannah (Bas-Saint-Laurent) said that she had the same seasonal job as her ex-Partner. Jenny (Îles-de-la-Madeleine) had to leave her job and her village because of harassment by her employer both before and after she quit her job. Geneviève (Bas-Saint-Laurent) could not meet the demands of her employer, who was also a member of her family.

It is important to note that many of the study participants were neither working nor looking for work at the time of their interviews. Louise and Danie (Îles-de-la-Madeleine) questioned whether they could ever go back to work, because both of them were over age 50 and had health problems. While all of the participants experienced very precarious living conditions and situations of homelessness, 12 participants (53 references) said that their remote locations and lack of transportation had made it harder to escape their violent situations and stabilize their living conditions.

by appearsIn the two regions considered in this study, homelessness appears directly related to problems in the local economy but is aggravated by the lack of social housing and the way that the local rental market disadvantages single women. Lack of low-income housing was mentioned by participants from several different parts of these two regions, and only two participants were living in social housing at the time of their interviews. These two women considered themselves very lucky to have secured such housing and believed that it had made all the difference in their ability to escape intimate partner violence. One of them, Louise, believed that lack of affordable housing keeps many women from leaving violent relationships:

I was lucky to get my housing. (…) Otherwise, I was in the street. (…) Because a lot of women endure the intolerable because they can’t leave. What let me leave was because I had that housing. (…) Renting, it's $800 for a small one-bedroom. And I don’t know their financial situations, but there is no housing. In (city name) everything is full, affordable housing is all full. There are waiting lists for two years, three years, five years. (Louise, Îles-de-la-Madeleine)

Housing costs appeared to be a vital issue for many of the participants (11 participants, 24 references), while others (seven participants, 25 references) stated that rents tended to be high in areas where the economy was dominated by traditionally male professions such as fishing, resource extraction, and manufacturing.

I have been (in a women's shelter) for 3 months now, but they had to extend it for another 3 months. I couldn’t find anything, I didn’t have affordable housing. I didn’t have an apartment. And an apartment in (city name) all alone, a two-bedroom, it's no lower than $700. And I couldn’t afford that! It's (because) of the wind turbines. Because of the turbines, you know, there are a lot of jobs, and that's why landlords raise the cost. It's because of that. (Suzie, Gaspésie)

Some participants also talked about the financial stress of seasonal employment.

Anne, who uses a wheelchair, told of her difficulties in getting out of a violent situation because of the lack of adapted housing in her region. She also faced additional costs for accessing services; for example, she had to pay for a 40-min paratransit trip whenever she neededd to take a bath.

Because for the last 5 years that I was there, I wanted to get out. But there was no way for me to get out. Because … well, being in a wheelchair, there's no accessible housing in the region. (Anne, Gaspésie)

Added to that was discrimination in housing, as Suzie described:

Apartments for women who are single parents, there should be some. You know, there are some, but they’re all taken, they’re rare. … You know, landlords, well then, you have a child, it's like it blocks everything. (Suzie, Gaspésie)

The local tourism industry also affects the housing market, particularly in Îles-de-la-Madeleine. As Danie explained:

There are a lot of houses for sale, but they’re not for rent. It's not for rent. If you rent a house in the fall, well you need to get out in the spring because of the tourists, they want to rent to tourists. So it's not always easy. (Danie, Îles-de-la-Madeleine)

Jenny, age 23 years, was working two jobs but still had to move back in with her parents. As she related:

I can’t leave because the cheapest house for sale is $75,000. … I can’t pay that by myself. There are no apartments, there's only some for tourists …, or you need to get out by the summer or else it costs $800 to $900. I can’t pay that. (Jenny, Îles-de-la-Madeleine)

Thus, among the women in our study, homelessness could be attributed in part to certain organizations and institutions that treated the violence that they had suffered as a private matter, and in part to conditions that impeded their social and economic integration and caused them significant housing instability and financial insecurity. Some participants who could not access second-stage shelter or social housing ended up going back to live with a violent spouse or other family member. Despite the suffering that they endured both from the violence itself and from their families’ reactions when they talked about it, some women were compelled to maintain ties with their partner or their immediate or extended families simply because they lacked the resources to secure adequate housing on their own.

### Representational Dimension: Traditional Roles and Maintaining Family Relationships at Any Cost

The participants’ comments (60 references) reflect the pervasiveness of stereotypical views about gender roles in rural areas, as supported by the results of research (DeKeseredy, 2019; Farhall et al., 2020; Savard & Marchand, 2016). Some of the women we interviewed raised the differences between urban and rural areas in the expression of gender roles and talked about how these differences had made their experiences more complex:

She always used to say to me: “Ah, we all know, a girl from the city, she's not like a girl from the country. She doesn’t like to clean,” and so on. Because for them, a woman is made to clean and cook and that's it, that's all. (Gisèle, Gaspésie)

Social responses to the violence experienced by the participants also appear to have been influenced by the Roman Catholic Church and particularly by its teachings on marriage and family. Louise offered a good example: “People no longer speak to me at church, because I got a divorce.” This comment illustrates the impacts that the expression of gender roles can have on women in rural settings and how it can weaken their support networks if they separate from their spouses (Farhall et al., 2020).

The emphasis on preserving the family unit, which seems more pronounced in rural areas (Clennett-Sirois, 2015; Farhall et al., 2020), also influenced some participants’ decisions to stay with an abusive spouse. They described their fear of displeasing friends and family by leaving their spouse, even though he was violent.

The following statement illustrates how traditional views of the couple and of the ideal family among women in more remote areas can make it harder for them to leave a violent spouse:

You know, I didn’t love him enough to want to do things with him anymore, things like that. But I still stayed, and I didn’t want to displease him. I didn’t want, like, to break up the family. But I didn’t love that man anymore. (Marie, Gaspésie)

Ambivalence and concern for maintaining family relationships are common to all women who are victims of intimate partner violence. But this study suggests that family and community values placed significantly greater pressure on our interviewees from two rural regions than on interviewees in other studies conducted in urban areas using similar methodology (Cousineau et al., 2021). Thus, because they live in such small communities—a concern expressed by 12 participants (53 references)—women in these two regions appear to be particularly subject to the judgment of others and to suffer from their disapproval, which can complicate their social and economic inclusion trajectory. The experiences of the participants in this study illustrate the losses suffered by those who try to resist this pressure or disapproval when, despite these costs, they permanently sever their ties with a partner or family member and thereby make their own living conditions more precarious.

Some participants also said that they had suffered the disapproval of their families because they had chosen a partner from another village or from an urban area. Other participants had strongly opposed the values about marriage and family that they were taught, thereby suffering the consequences and the many losses associated with their break-up. Faced with the harsh living conditions that they would suffer if they left, other participants had to resign themselves to maintaining their family ties or continuing to live with their abusers or with people who had shielded them. Forced to live with her family, Jenny (Îles-de-la-Madeleine) described this sense of resignation, which she attributed to the values that had been passed on to her:

And there's also the fact that it's family. And in (name of town) family … ahh that is one of the values. Family, we must always love our family. We always need to be close to our family. Family are your only friends that you will have for your entire life. Your family are the only people that will be there for you your entire life. Not just the mom-and-dad family, but also brothers, sisters, cousins, and grandma and grandpa, aunt and uncle. Uncle. Whatever. Uncle through marriage, I don’t have other uncles. Even when you know that your family hurts you more than anything. … But you don’t have the choice but to love them, and you don’t have the choice but to live with them. (Jenny, Îles-de-la-Madeleine)

## Discussion

This study showed how family, friends, and certain institutions and organizations questioned and denied reports of intimate partner violence from women living in two rural regions of Quebec, suppressing their voices and leaving them to suffer in private. For many of the women we interviewed, disclosing intimate partner violence weakened their relationships within their primary support networks, jeopardized their access to safe, stable housing, and complicated their access to formal and informal emotional and psychological support. As a result of all these circumstances, combined with a local labor market that favors men and special challenges that single women face in accessing shelters, social support, and suitable, affordable housing, many of these women became homeless or were forced to live with their abusers or with relatives who had shielded them.

This entire set of conditions can be seen as a product and a reaffirmation of White male privilege in non-urban settings. DeKeseredy et al. (2016, 2019) have previously observed how a culture of masculinity complicates the journey and experiences of women who are victims of violence in such settings. The present study adds to this research by showing that these issues pervade the organization and structure of these communities, reducing opportunities for abused women to stabilize their living conditions, obtain help, and access justice, especially when they attempt to disclose or escape from male violence.

### A Social and Economic Structure That Perpetuates Male Domination

In geography and urban studies, a number of theories that spatialize the materialistic analysis of social relations reveal the inequalities between urban centers and outlying non-urban settings (Bouchard, 2013; Brenner & Schmid, 2015). In particular, government policies tend to encourage such rural regions to invest in and organize their economies around extracting natural resources or processing them into materials that meet the needs of large urban centers (Arboleda, 2016). Both materialistic feminism and the intersectional framework recognize the intersection between the organization of a capitalist economy and the perpetuation and reproduction of a patriarchal system. The information provided by the women in this study shows that the two regions where they live are no exception. Their economies are structured mostly around the fishing industry and the manufacturing of wind turbines. These industries place men who work for them in a position of privilege that tends to afford them some protection against accusations of conjugal or family violence. In addition, the structure of these regions’ economies drives up the cost of living in general, and housing rents in particular. The episodes of homelessness experienced by women in this study and the significant economic inequalities that persist between men and women in these regions (SCF, 2015) testify to the ways that this structural arrangement disadvantages women. In addition, in the Îles-de-la-Madeleine region, homeowners can charge high rents to tourists in the summertime, placing women who rent and live alone at a disadvantage and making their living conditions even more precarious and their housing even more unstable in that season. Research shows that many women facing homelessness will go back to live with intimate partners even if they are violent (Flynn et al., forthcoming). So these unfair conditions in the housing market may place women in situations where they are likely to be assaulted again. These structural processes also limit women's independence from their partners and their families, forcing them to conform to their subordinate role in social institutions such as the couple or the family.

Applying a variety of conceptual frameworks, the intersectional analysis presented in this article focuses on the various levels at which and areas in which social power relations can occur and recur. Although our sample did include one Indigenous woman and one trans woman, we were not able to assess how colonialism and cisgenderism specifically structured and impacted their life courses. This is the most critical limitation of this study and may have contributed to making these women's specific experiences invisible, so that the knowledge produced here concerns the White, cisgender majority of women only. This limitation may be attributable to our recruitment strategy, which we deployed mainly in community organizations. Given that the silencing of women's voices and their withdrawal into the private sphere were central concerns of the present study, we can hypothesize that BIPOC women at the crossroads of social power relations that give them less privilege than the women who participated in this study are likely to be invisible even in these community organizations. Future studies on women's homelessness should diversify their recruitment strategies to reach these underrepresented groups, then analyze their specific experiences. Such research is all the more urgent because organized identity groups are currently broadcasting racist speech and hate propaganda in various regions of Quebec (Potvin, 2017), which is likely to perpetuate the hegemony of White masculinity. Although this study did not apply critical anti-racist theory in its intersectional analysis, we must nevertheless emphasize the whiteness of the culture and the masculinity of the structures discussed here. We must also underscore that the precarious living conditions, poverty and homelessness into which the participants were driven when they tried to escape male violence were reinforced by the sexual division of labor within the capitalist system and in some cases by its interaction with ableism or ageism.

## Conclusion: Excluding and Punishing Abused Women to Maintain the Status quo

Ultimately, the social responses presented in this article are what Romito (2008) would describe as tactics that suppress speech and subversion by women and reinforce domination by men. For example, participants in this study who questioned or drew attention to male power in the community found themselves punished or relegated to subordinate, less privileged roles and social positions. Certain institutions in the two regions studied, including the police, welfare services, the Roman Catholic Church, and even some workplaces, have been vectors for women's withdrawal from the public space. Our interviews with Jenny, Hannah, and Geneviève, all of whom were forced to leave their jobs, show how the protection that the community affords abusers can contribute to women's impoverishment and their disappearance from the workplace. Within our sample, none of the participants who were older or living with disabilities had high economic status, which is consistent with the known impacts of ageism and ableism. Police in these regions frequently fail to treat intimate partner violence as a public matter. In Joannie's case, police involvement led to her going to jail, while in Diana's, lack of services and the exhaustion of available resources led her to placement in a psychiatric institution.

This study has shown the association between violence against women and homelessness among women in sparsely populated rural communities. It has shed new light on how social responses to violence in such communities can insidiously produce, reproduce, or perpetuate a culture and structure of masculinity and, more broadly, help force women back into subordinate roles in the private sphere. This dynamic increases their risk of becoming homeless or experiencing economic and social insecurity. Given the many losses and high price paid both by women who strongly oppose this dynamic and by those who resign themselves to it, further research must be done to document the experience of women who try to overturn or emancipate themselves from such power relations in such communities. From a policy and practice perspective, it is essential to consolidate and better support the network of assistance services for abused women in rural regions and to develop a broader strategy to advocate for greater equality with urban regions, facilitating autonomy and social participation for all women. This strategy must include policy-supported initiatives that enable women to break out of the domestic and reproductive roles to which they are consigned by the capitalist system, especially in regions where the exploitation of natural resources is made possible by the subordination of women (Robert, 2017). The political emancipation of women and the reduction of violence against them also require in-depth transformations that let us think beyond gender roles and deconstruct White male entitlement so as to change the distribution of power in regions with resource-based economies.
